# Method of Concentrating Entamoeba Cysts in Stools

**Published:** 1918-05

**Authors:** 


***Method of Concentrating Entamoeba Cysts in Stools.*** By J. W. Cooper, and F. W. Harold Row. Abs. from *The Lancet,* Feb. 3, 1917.

A lump of fæces of at least 1 gramme in weight is shaken up with about 30 cc. of normal saline (0.8 o/o solution of Na Cl) per gramme of fæces to disintegrate the mass into individual particles and form an emulsion which settles very slowly. The emulsion is separated and shaken up with ether. The fecal débris absorbing ether become lighter and remain at the top, whereas the cysts, which do not absorb ether, remain at the bottom.

The time allowed for mechanical shaking is extremely long, for otherwise the cysts would not appear in the final deposit.

For emulsifying the fæces saline is preferable.

In an ordinary formed stool at least 90 o/o of the weight of fæces employed is extracted.

The period found most suitable for centrifuging with 15 cc. tubes is either 1 1/2 minutes at 1200 revolutions per minute, or 2 1/2 minutes at 600 revolutions.

As ether might have a bad effect upon the cysts, the author adopted a method requiring no chemicals.

Ten grammes of fæces are shaken with 100 grammes of normal saline. The emulsion obtained is filtered; the filtrate is then centrifuged and the supernatant liquid poured off. The volume is made up again with normal saline. This process is repeated several times, until the supernatant liquid is almost clear. Loopfuls of the deposit are used to make hanging-drop preparations for culture experiments.

